# Experimental Inquiry Concerning the Question, Whether the Purgative Action of the Neutral Salts Is the Effect of Endosmosis?

**Published:** 1853-03

**Authors:** H. Aubert


*Experimental Inquiry concerning the question, whether the Purgative Action of the Neutral Salts is the effect of Endosmosis?* By Dr. H. Aubert.—Aubert made his experiments with the purpose of examining the correctness of the view spread under *Liebig's* authority ("Untersuchung der Mineralquelle zu Soden und Bemerkungen ueber die Wirkung der Salze auf den Organismus"—*Wiesbaden*, 1839), that the purgative action of the neutral salts is a merely physical process, being the consequence of exosmotical transudation from the walls of the intestinal tube, effected by the more concentrated solution of these salts within the cavity of the tube. The experiments were instituted in the following manner:—Solutions of different neutral salts were put into a cylindrical glass tube, the lower end of which was covered with a piece of membrane, from a pig's bladder; then the tube was immersed into serum of blood, and the changes going on in the solution within, and the serum without, were examined at different intervals. Another series of experiments were made by taking internally solutions of these salts. As the result of both series, Aubert draws the following inferences:

1. The purgative effect is not influenced by the degree of concentration of the solution; the number of stools produced by a certain quantity of salt will be the same, whether the salt is dissolved in six or seventytwo ounces; the water of the solution is excreted by the kidneys; the salt exercises its influence on the bowels. 2. No albumen is found in the alvine excretions, as ought to be the case, if the action of the salts was an endosmotical one. 3. The quantity of salt excreted through the urine, compared with the quantity of water contained in the discharge from the bowels, is not that which it ought to be, according to the laws of endosmosis and exosmosis (as much, at least, as they are known at present). 4. Peristaltic motion of the bowels is constantly excited by the neutral salts; the rolling and rumbling, which Aubert always observed soon after taking the salts, as well in a concentrated as when in a diluted solution, is attributed by him to the action of the salts on the nerves of the intestines, and to the reflex motion excited in consequence of this. 5. A part of the *sulphate of magnesia* appears to be decomposed within the organism, as the magnesia is excreted in a larger proportion than the sulphuric acid with the fæces; the sulphuric acid in a larger one than the magnesia with the urine. 6. The remedy produces the characteristic effect on the bowels, when a solution of it is merely infused into the veins of an animal.

It must be, however, remarked here, that *Liebig* himself, in a work of a later date, (" Untersuchungen ueber einige Ursachen der Saeftebewegung im thierischen Organismus," 1848,) states, that he does not intend to explain the whole action of the neutral salts by endosmosis, but that he considers this to be one of their influencing qualities.—*Ibid*, *from Henle und Pfeufer's Zeitschr. fur Ration. Medic.*

